# Molecular Interaction of Soluble Klotho with FGF23 in the Pathobiology of Aortic Valve Lesions Induced by Chronic Kidney Disease

**DOI:** 10.7150/ijbs.92447

**Published:** 2024-06-17

**Authors:** Erlinda The, Yufeng Zhai, Qingzhou Yao, Lihua Ao, David A. Fullerton, Xianzhong Meng

**Affiliations:** Departments of Surgery and Medicine, University of Colorado Denver, Aurora, CO 80045, USA.

**Keywords:** Chronic kidney disease, calcific aortic valve disease, fibroblast growth factor 23, Klotho, YAP

## Abstract

Chronic kidney disease (CKD) is linked to greater prevalence and rapid progression of calcific aortic valve disease (CAVD) characterized by valvular leaflet fibrosis and calcification. Fibroblast growth factor 23 (FGF23) level is elevated, and anti-aging protein Klotho is reduced in CKD patients. However, the roles of FGF23 and Klotho in the mechanism of aortic valve fibrosis and calcification remain unclear. We hypothesized that FGF23 mediates CKD-induced CAVD by enhancing aortic valve interstitial cell (AVIC) fibrosis and calcification, while soluble Klotho inhibits FGF23 effect.

**Methods and Results:** In an old mouse model of CKD, kidney damages were accompanied by aortic valve thickening and calcification. FGF23 levels in plasma and aortic valve were increased, while Klotho levels were decreased. Recombinant FGF23 elevated the inflammatory, fibrogenic, and osteogenic activities in AVICs. Neutralizing antibody or shRNA targeting FGF23 suppressed the pathobiological activities in AVICs from valves affected by CAVD. FGF23 exerts its effects on AVICs via FGF receptor (FGFR)/Yes-associated protein (YAP) signaling, and inhibition of FGFR/YAP reduced FGF23's potency in AVICs. Recombinant Klotho downregulated the pathobiological activities in AVICs exposed to FGF23. Incubation of FGF23 with Klotho formed complexes and decreased FGF23's potency. Further, treatment of CKD mice with recombinant Klotho attenuated aortic valve lesions.

**Conclusion:** This study demonstrates that CKD induces FGF23 accumulation, Klotho insufficiency and aortic valve lesions in old mice. FGF23 upregulates the inflammatory, fibrogenic and osteogenic activities in AVICs via the FGFR/YAP signaling pathway. Soluble Klotho suppresses FGF23 effect through molecular interaction and is capable of mitigating CKD-induced CAVD.

## Introduction

Chronic kidney disease (CKD) is a growing global health concern, with its incidence rising significantly as the population ages. Among older adults in the United States, CKD has a prevalence of 39.4% 1. CKD is a significant contributor to the morbidity of many diseases, including cardiovascular diseases 2,3.

Calcific aortic valve disease (CAVD) is the most common heart valve disease and ranks as the third most prevalent cardiovascular disease 4. According to the Global Burden of Disease Study 2019, the incidence, prevalence, and deaths associated with CAVD increased over the last three decades 5. In the United States alone, CAVD affects 13% of those over 65 years old, and the incidence rises further with age 6. Given the rising number of older people in the United States, this will increase the healthcare burden. Unfortunately, there are currently no effective pharmacological treatments for CAVD, with surgical or interventional valve replacement being the only viable option 7. The prevalence of CAVD increases as the glomerular filtration rate declines; subsequently, CKD is a significant risk factor for CAVD 8. However, the mechanism underlying CKD-induced CAVD remains unclear. As such, there is an urgent need to develop pharmacotherapeutic approaches for prevention of CAVD progression.

Aortic valve interstitial cells (AVICs) are the predominant cells in aortic valve leaflets and play a major role in the pathogenesis of CAVD 9,10. Fibroblast growth factor 23 (FGF23), a protein mainly produced by osteoblasts and osteocytes 11, has been shown to induce airway epithelial cells to release interleukin 1β 12. As a pro-inflammatory cytokine, interleukin 1β is capable of inducing inflammatory and osteogenic responses in AVICs 13,14. Several studies demonstrate that FGF23 plays a role in mediating inflammation associated with CKD 15,16. While plasma level of FGF23 increases progressively in subjects with CKD 17, the role of FGF23 in aortic valve lesions associated with CKD remains unclear.

Klotho, a membrane-bound and soluble anti-aging protein, has anti-inflammatory properties 18. Klotho is predominantly expressed in the kidney, and its levels are lower in calcified aortic valve tissue 19. Recombinant Klotho downregulates AVIC inflammatory responses to the soluble, extracellular protein matrilin-2 that functions as a damage-associated molecular pattern 10. Reduced levels of Klotho in CKD patients are accompanied by increased levels of FGF23 20-22. Soluble Klotho has been found to suppress FGF23-mediated NF-κB activation 23. While membrane Klotho is a co-receptor for FGF23 24, soluble Klotho may function as an FGF23 antagonist. Investigation of the mechanism underlying the interaction of soluble Klotho with FGF23 may lead to the development of therapeutic approaches for the treatment of CAVD in CKD patients.

In this study, we tested the hypothesis that FGF23 enhances AVIC fibrosis and calcification, and soluble Klotho inhibits the effect of FGF23 on aortic valve fibrosis and calcification induced by CKD. The objectives of this research are to determine: (1) the effect of FGF23 on AVIC inflammatory, fibrogenic and osteogenic activities, (2) the mechanism by which FGF23 induces valvular cell fibrosis and calcification and (3) whether soluble Klotho inhibits FGF23-induced AVIC fibrosis and calcification through interaction with FGF23.

## Materials and Methods

### Chemicals and reagents

Endotoxin-free recombinant human FGF23 (2604), human Klotho (MAB5334), mouse Klotho (1819), neutralizing antibody against FGF23 (AF2604), and antibodies against YAP (867711) were purchased from R&D Systems, Minneapolis, MN. Antibody against intercellular adhesion molecule 1 (ICAM-1, SC8439) was acquired from Santa Cruz Biotechnology, Dallas, TX. Antibodies against runt-related transcription factor 2 (RUNX2, DIH7) and vascular cell adhesion molecule 1 (VCAM-1, EIE8X) were obtained from Cell Signaling Technology, Danvers, MA. Antibodies against collagen IV (ab6586), alkaline phosphatase (ALP, ab108337), FGF receptor (FGFR) 1 (ab10646) and FGFR4 (ab44971) were purchased from Abcam, Cambridge, UK. Creatinine assay kit (MAK080), phosphate assay kit (MAK030), Tween-20 (P7949) and collagenase (C5138) were acquired from Sigma-Aldrich, St. Louis, MO. Antibodies against FGF23 (PA5-101323) and Klotho (PA5-88303), and medium 199 (11150067) were purchased from Thermo Fisher Scientific, Waltham, MA. Antibody against collagen I (PA2140-2) was purchased from Boster Bio, Pleasanton, CA. Verteporfin (5305) was purchased from Tocris Bioscience, Bristol, UK. PRN1371 (S8578), ponatinib (S1490) and BLU-554 (S8503) were purchased from Selleck Chemicals, Houston, TX.

### Animals and treatment

All experimental protocols were approved by the Institutional Animal Care and Use Committee of the University of Colorado Denver. This study complied with the NIH guidelines for the Care and Use of Laboratory Animals (National Research Council 1996).

Older adults exhibit greater incidence and severity of CKD, resulting in higher prevalence of CAVD in this population. Older CKD patients have reduced nephron count and glomerular filtration rate. We used old C57BL/6 mice (18-20 months) to establish a CKD model mimicking pathological changes observed in older adults with CKD.

The old mouse CKD model was developed by feeding an AIN-76A diet (S1515, Bio-Serv, Flemington, NJ) to animals in the 6-week experiment period. Oral gavage was used to administer calcitriol (0.25 μg/kg) and adenine (100 μL) from week 3 to 5. Sham mice were fed an AIN-76A diet from week 0 to 6 and administered phosphate-buffered saline (PBS, 100 μL) orally via gavage from week 3 to 5. For Klotho treatment, CKD-developing mice were implanted subcutaneous osmotic pumps to constantly deliver recombinant Klotho (20 µg/kg/day) from week 3 to 6. Control mice were treated with PBS using subcutaneous osmotic pumps. All mice were sacrificed after 6-week experiment.

### Isolation and culture of human AVICs

Normal aortic valves (aortic valves with a normal morphology) were obtained from the explanted hearts of heart transplant patients diagnosed with cardiomyopathy. Diseased aortic valves were collected from patients with aortic stenosis who underwent aortic valve replacement at the University of Colorado Hospital. The study was approved the Institutional Review Board of the University of Colorado Denver and adhered to the principles outlined in the 1964 Declaration of Helsinki, with revisions in 2013. Written informed consent was obtained from all donors.

Valvular tissue was washed with PBS. The tissue was then cut into 1-2 mm fragments and subjected to treatment with collagenase type I for 30 minutes at 37°C to eliminate endothelial cells. Tissue digestion continued using a fresh collagenase solution at 37°C for 4-6 hours. Subsequently, cell suspensions were centrifuged for 10 minutes at 1,000 rpm, and the resulting cell pellets were resuspended and cultured in medium 199 supplemented with 10% fetal bovine serum (FBS) and 1% Penicillin/Streptomycin within a 37°C humidified incubator containing 5% CO2.

During both the growth and experimental phases, culture media were refreshed every 3 days. AVICs from passages 3 to 6, reaching 80-90% confluence, were used in all experiments, and data were collected from 10 distinct donor sources. To evaluate the impact of FGF23 on AVIC inflammatory, fibrogenic and osteogenic activities, cells were exposed to recombinant FGF23 (40 ng/mL) or a neutralizing antibody against FGF23 (10 μg/mL) for a duration of 72 hours. Cell lysates were then utilized to quantify ICAM-1, VCAM-1, collagen I, collagen IV, RUNX2 and ALP proteins. To investigate the mechanism by which FGF23 exerts its effects on AVICs, we pre-treated cells with PRN1371 (a global FGFR inhibitor), ponatinib (a relatively selective FGFR1 inhibitor at a low concentration), BLU-554 (a FGFR4 inhibitor), verteporfin (a YAP inhibitor) or recombinant Klotho before an expose to FGF23.

### Histology

Kidney and aortic valve tissues were embedded in optimal cutting temperature compound and cryopreserved into 5 μm-thick sections. Subsequently, sections were fixed in 4% paraformaldehyde for 15 minutes. Hematoxylin and eosin (H&E) staining was conducted to analyze tissue morphology. For evaluation of calcium deposits within aortic valve tissues, Von Kossa staining was carried out by the Histology Core at the University of Colorado Denver. Microscopic examination was performed using a Nikon microscope.

### Immunofluorescence staining

Aortic valve tissue sections from both sham and CKD mice were subjected to permeabilization using a mixture of 70% methanol and 30% acetone, followed by fixation with 4% paraformaldehyde for 15 minutes at room temperature. To minimize non-specific antibody binding, tissue sections were blocked with 10% goat serum for 30 minutes at room temperature. Subsequently, the tissue sections were incubated with antibodies against FGF23 (dilution: 1:100) or against Klotho (dilution: 1:50) overnight at 4°C. Following thorough washing with PBS, the tissue sections were subjected to incubation with Cy3-conjugated secondary antibodies for 2 hours at room temperature. Nuclei were counterstained with 4',6-Diamidino-2-phenylindole (DAPI). Additionally, Alexa 488-tagged wheat germ agglutinin (WGA) was employed to delineate the overall tissue structure. Microscopic examination was performed using a Leica CTR5500 digital microscope (Leica Mikroskopie und Systeme GmbH, Wetzlar, Germany).

### Picrosirius red (PSR) staining

The PSR staining is specifically designed to identify collagens and serves as a valuable tool for evaluating fibrogenic activity in cultured cells. In the experiments for *in vitro* fibrogenesis, AVICs were exposed to FGF23 for 14 days. Subsequently, AVICs underwent overnight treatment with methanol at -20°C. Cells were then incubated in 0.1% PSR for 4 hours and were rinsed with 0.1% acetic acid, air-dried, and observed under a microscope. To elute the color for quantitation, cells were treated with 0.1 ml of 0.1 M sodium hydroxide for 2 hours at room temperature. The optical density of the collected solution was measured at 540 nm using a spectrophotometer (BioTek Instruments, Inc., Winusky, VT, USA).

### Alizarin red S staining

Alizarin Red S staining (A5533, Sigma-Aldrich, St. Louis, MO) was employed to detect calcium deposition in the AVIC culture. The cells were incubated in pro-calcification media, comprising medium 199 supplemented with 10 nmol/L of dexamethasone, 10 mmol/L of β-glycerophosphate, 8 mmol/L of calcium chloride and 4.0 μg/mL of cholecalciferol for a duration of 14 days in the presence or absence of FGF23. Cells were fixed using 4% paraformaldehyde and were subsequently incubated with a 0.2% Alizarin Red S solution (pH 4.0-4.2) for 30 minutes. Excess dye was removed by thoroughly rinsing with distilled water. For quantification purposes, the stain was rinsed off using 10% acetic acid at 75 °C and subsequently measured using a spectrophotometer at a wavelength of 450 nm.

### RNA extraction and RNA sequencing

RNA samples were submitted to Novogene Co. (Sacramento, CA, USA) for a thorough quality control assessment, including evaluations of RNA purity, integrity and quantification. Nanodrop, Qubit and Agilent 2100 instruments were employed for these assessments. Additionally, Novogene Co. prepared RNA-Seq libraries from the total RNA through poly(A) enrichment and performed sequencing on the Illumina Novaseq 6000 PE150 platform.

### FGF23 and Klotho knockdown

Lentiviral shRNA targeting human FGF23 (TRCN0000058765), human Klotho (TRCN0000158579) and the scramble shRNA (pLKO.1-puro non-target) were obtained from the Functional Genomics Core Facility at the University of Colorado Denver. AVICs at 80% confluence underwent lentiviral transduction with vectors carrying scramble or FGF23 shRNA or Klotho shRNA, with subsequent selection using 1 μg/mL puromycin (A1113803, Thermo Fisher Scientific, Waltham, MA).

### Kidney function assay

Plasma levels of creatinine and phosphate were assessed using assay kits (Sigma-Aldrich, St. Louis, MO) following the manufacturer's instructions. An automatic microplate reader (Biotek, Winooski, VT) was used to analyze samples and standards in triplicate at a wavelength of 570 nm (for creatinine) and 650 nm (for phosphate).

### Immunoblotting

Protein levels of ICAM-1, VCAM-1, collagen I, collagen IV, RUNX2, ALP, FGFR1, FGFR4 and YAP were analyzed using immunoblotting. Laemmli sample buffer (161-0737, Biorad, Hercules, CA) was used to lyse AVICs. Subsequently, the samples underwent electrophoresis on 4-20% SDS-PAGE gels. The proteins were transferred onto nitrocellulose membranes. Following blocking with 5% skim milk for 1 hour at room temperature, membranes were incubated overnight incubation at 4°C with primary antibodies (diluted within the range of 1:200 to 1:500). Horseradish peroxidase-linked secondary antibodies (diluted at 1:10,000) were applied, and protein bands were visualized using enhanced chemiluminescence reagents. β-actin was utilized as an internal control for sample loading. Band quantification was performed using Bio-Rad's ImageLab software (Hercules, CA).

To examine Klotho and FGF23 interactions, we incubated recombinant FGF23 with recombinant Klotho for 24 hours at 37°C. Then samples of the protein mixture were electrophoresed on native gels. The proteins were transferred onto nitrocellulose membranes. Following blocking with 5% skim milk for 1 hour at room temperature, membranes were incubated overnight incubation at 4°C with antibody against FGF23 or Klotho. Then, the membranes were processed using the method described above for visualization of bands corresponding free FGF23 and FGF23/Klotho complex on membrane probed using anti-FGF23, as well as free Klotho and FGF23/Klotho complex on membrane probed using anti-Klotho.

### Statistical analysis

Statistical analysis was conducted to evaluate the significance of differences using various methods. An unpaired, two-tailed Student's t-test and/or one-way ANOVA with Tukey's multiple comparisons test was carried out using GraphPad Prism 9 (GraphPad Software, San Diego, CA). Additionally, a nonparametric Mann-Whitney U test was employed to validate differences between the two groups being compared. In cases of multiple group comparisons, Tukey's test was used to confirm differences. Statistical significance was defined as a *P* value ≤ 0.05. Data are presented in the format of mean ± standard error of the mean (SEM).

## Results

### FGF23 levels are increased while Klotho levels are decreased in CKD mice

**Figure 1:**
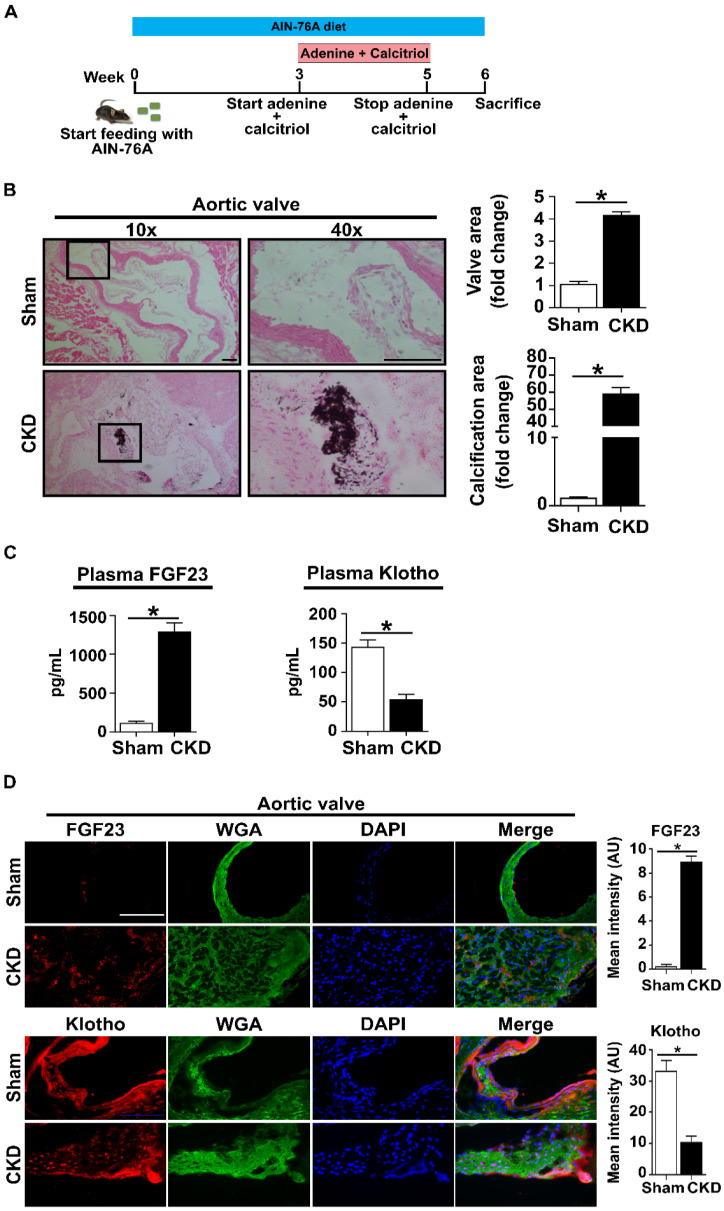
** CKD increases FGF23 levels and decreases Klotho levels in the plasma and aortic valves of old mice. (A)** Schematic diagram outlining the experimental protocols. Old mice (18-20 months) were on AIN-76A diet for 6 weeks and subjected to treatment with adenine and calcitriol from week 3 to week 5.** (B)** Representative images of von Kossa staining show valvular thickening and calcified nodule formation in the aortic valves of CKD mice. Scale bar = 100 μm. **(C)** CKD mice have higher FGF23 levels and lower Klotho levels in plasma in comparison to sham controls. **(D)** Representative images of immunofluorescence staining show increased FGF23 levels and reduced Klotho levels in aortic valves of CKD mice. Original magnification 40x. Scale bar = 100 µm. Quantitative data are expressed as mean ± SEM. n = 4 mice per group. *P < 0.05 versus sham controls.

To assess the impact of CKD on the aortic valve, we established a CKD model using old C57 mice (18-20 months old) fed with AIN-76A diets and treated with adenine and calcitriol (**Figure 1A**). The kidneys of CKD mice are smaller and appear pale when being compared to the kidneys in sham-treated mice (**Supplementary Figure 1A**). Histological examination and immunostaining revealed tubular damage and monocyte/macrophage accumulation in the kidney tissue of CKD mice (**Supplementary Figure 1B** and** C**). Plasma creatinine and phosphate levels are markedly increased in CKD mice (**Supplementary Figure 1D** and** E**). In addition, Klotho protein levels are decreased in the kidneys of CKD mice compared to those of sham mice (**Supplementary Figure 2**). Aortic valve lesions, including thickening and calcification, are evident in CKD mice (**Figure 1B**). Furthermore, plasma and valvular tissue levels of FGF23 are markedly increased in CKD mice, while plasma and valvular Klotho levels are markedly decreased (**Figure 1C** and** D**).

### FGF23 elevates the inflammatory, fibrogenic and osteogenic activities in AVICs

**Figure 2:**
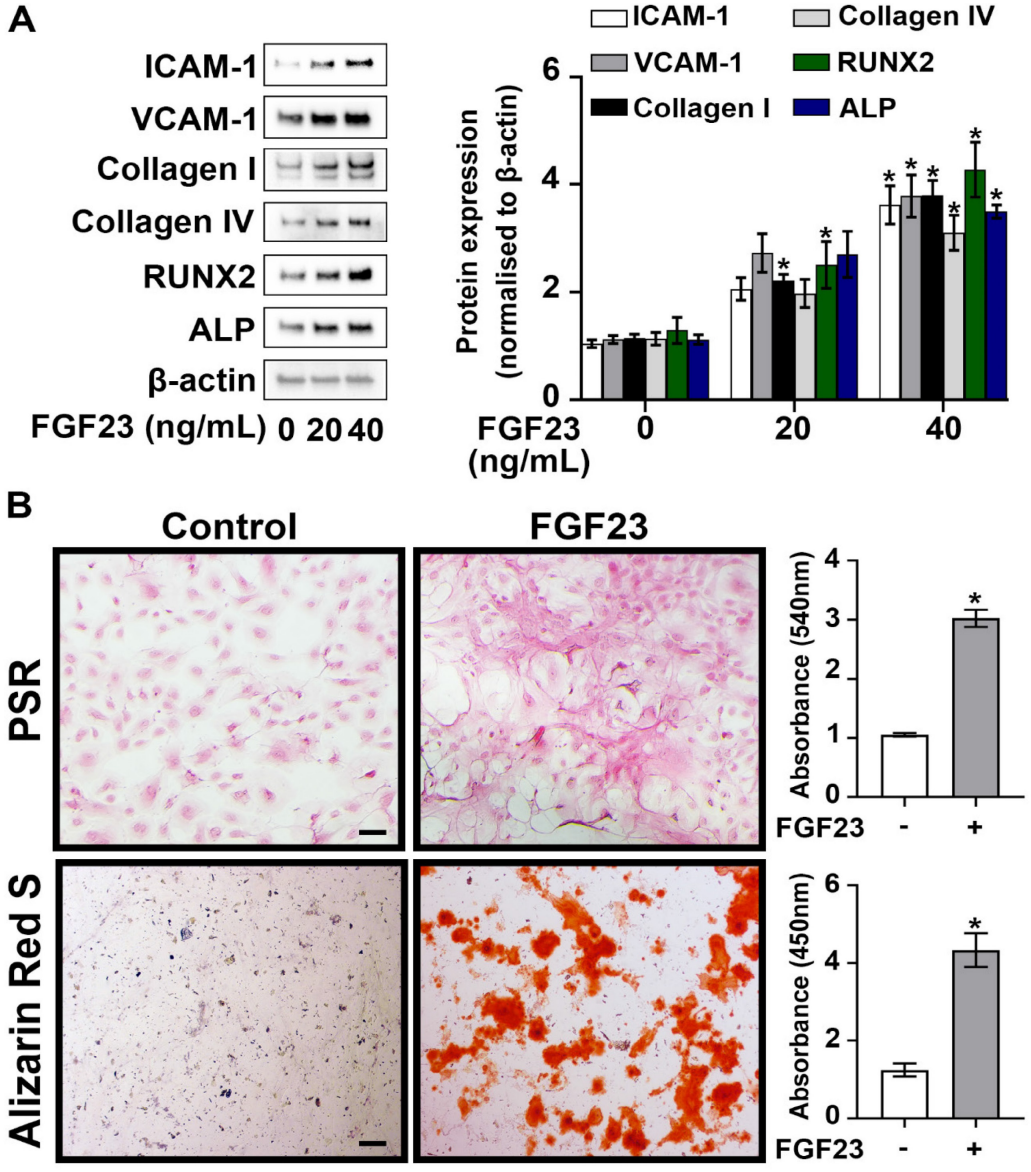
** FGF23 induces AVIC fibrosis and calcification. (A)** Human AVICs from normal valves were treated with recombinant FGF23 in different concentrations for 72 hours. Representative immunoblots (left) and densitometric data (right) show that FGF23 upregulates the expression of inflammatory (ICAM-1 and VCAM-1), fibrogenic (collagen I and collagen IV) and osteogenic (RUNX2 and ALP) mediators in AVICs. **(B)** Representative images of Picrosirius Red (PSR) staining (upper) and Alizarin Red S staining (lower), along with corresponding spectrophotometric data, show that prolonged treatment with recombinant FGF23 (10 days or 14 days) induces collagen and calcium deposition in AVICs. Images were taken using a 10x objective. Scale bar = 100 µm. Quantitative data are expressed as mean ± SEM, n = 4 cell isolates from distinct donor valves in each group. *P < 0.05 versus untreated control.

Given the higher circulating levels of FGF23 observed in CKD mice, we investigated the impact of FGF23 on AVIC pathobiology associated with CAVD. Cultured normal human AVICs were exposed to recombinant FGF23 at concentrations of 0, 20, and 40 ng/mL for 72 hours. As shown in **Figure 2A**, treatment with recombinant FGF23 (40 ng/mL) significantly upregulated the expression of ICAM-1, VCAM-1, collagen I, collagen IV, RUNX2, and ALP. PSR staining revealed a significant increase in collagen deposition in AVICs exposed to FGF23, and Alizarin Red S staining documented greater calcium deposition and nodular calcification in AVICs exposed to FGF23 (**Figure 2B**). These findings collectively underscore the potent effect of FGF23 in elevation of inflammatory, fibrogenic and osteogenic activities in human AVICs.

### FGF23 and Klotho are negatively correlated

**Figure 3:**
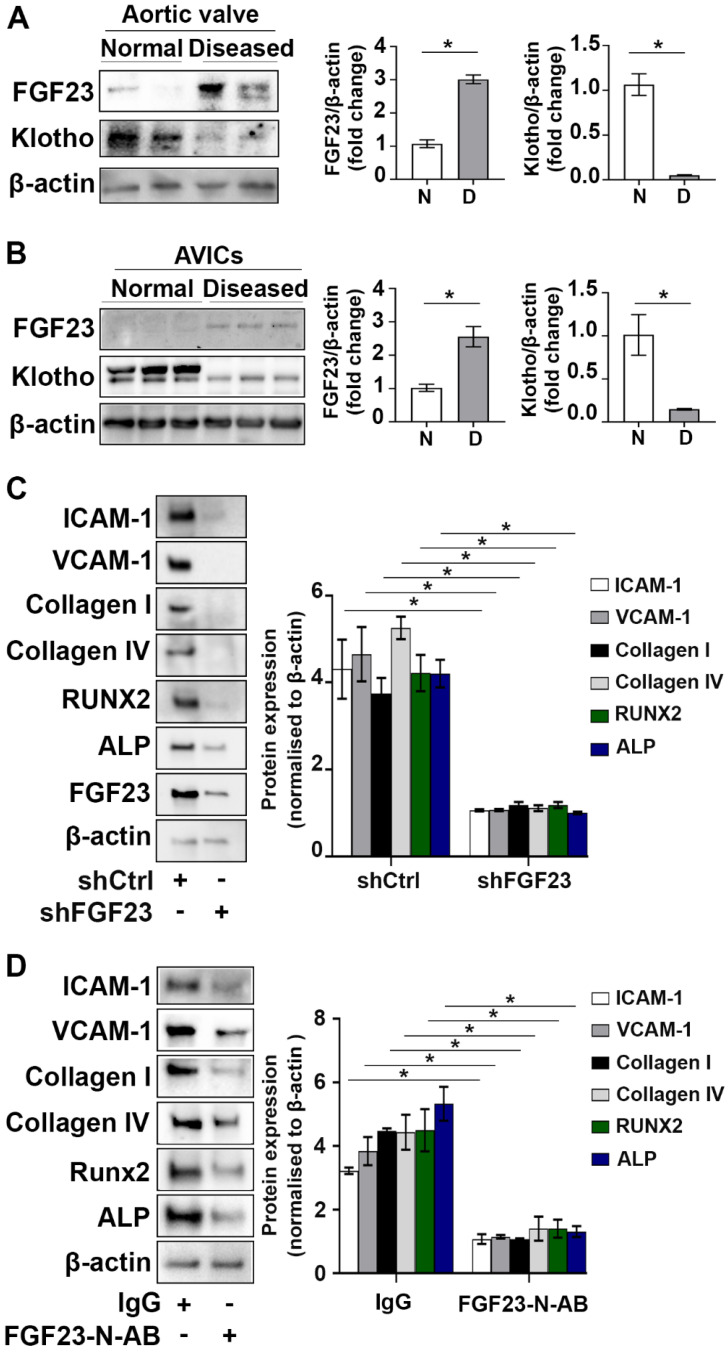
** FGF23 is involved in mediating the pathobiological activities in AVICs of CAVD valves. (A)** Immunoblots and densitometric data show that calcified aortic valves from patients with CAVD have higher levels of FGF23 and lower levels of Klotho in comparison to normal aortic valves. Values are means ± SEM. n = 4 aortic valve tissues from distinct donor in each group; *P < 0.05 versus normal aortic valves. N = normal. D = diseased. **(B)** Immunoblots and densitometric data show that human AVICs from diseased aortic valves have increased levels of FGF23 and decreased levels of Klotho. Values are means ± SEM. n = 4 cell isolates from distinct donor valves in each group. *P < 0.05 versus cells from normal valves.** (C** and** D)** Human AVICs from diseased valves were treated with lentivirus expressing FGF23 shRNA (100 nmol/L) or FGF23-neutralizing antibody (5 µg/mL) for 72 hours. Representative immunoblots and densitometric data show that AVICs treated with FGF23 shRNA or FGF23-neutralizing antibody have reduced levels of inflammatory, fibrogenic and osteogenic mediators. Values are mean ± SEM, n = 3 cell isolates from distinct donor valves in each group. *P < 0.05 versus control. Ctrl = control. N-AB = neutralizing antibody.

As shown in **Figure 3A** and** B**, FGF23 levels were significantly increased in human aortic valve tissues from valves affected by CAVD and AVICs isolated from diseased valves. In contrast, Klotho levels were decreased in diseased valve tissues and cells. To examine whether excessive FGF23 level plays a role in enhancing valvular fibrosis and calcification, we conducted gene knockdown of FGF23 in AVICs from diseased valves and assessed the impact of FGF23 knockdown on the expression of inflammatory, fibrogenic and osteogenic mediators. Reduction of FGF23 level in AVICs of diseased valves suppressed spontaneous inflammatory, fibrogenic and osteogenic activities (**Figure 3C**). In addition, FGF23-neutralizing antibody had similar effects in suppressing the spontaneous inflammatory, fibrogenic and osteogenic activities in AVICs of diseased valves (**Figure 3D**). Together, these data demonstrate that FGF23 and Klotho are negatively correlated in aortic valve and valvular cells, and downregulation of FGF23 reduces the spontaneous fibrogenesis and calcification in AVICs of diseased aortic valves.

### FGF23 induces AVIC fibrosis and calcification via the FGFR/YAP signaling pathway

**Figure 4:**
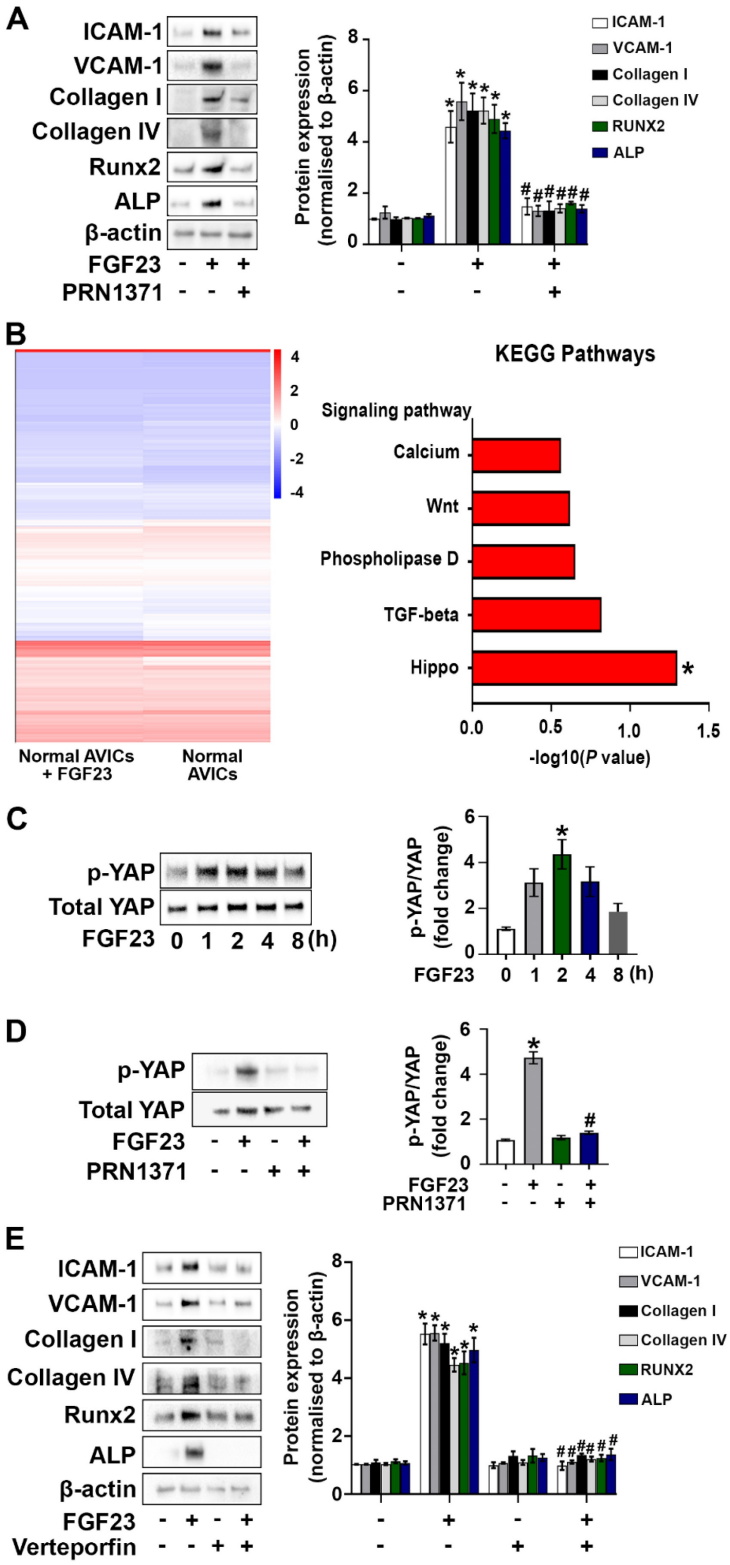
** FGF23 upregulates AVIC pathobiological activities via activation of the FGFR/YAP signaling pathway. (A)** Human AVICs from normal valves were pre-treated with global FGFR inhibitor PRN1371 (1.0 µM) for 2 hours, followed by treatment with FGF23 (40 ng/mL) for 72 hours. Representative immunoblots and densitometric data show that FGF23 induces the production of inflammatory, fibrogenic and osteogenic mediators in AVICs via FGFR. **(B)** Total RNA-sequencing was applied to analyze gene expression in AVICs incubated in the presence or absence of recombinant FGF23. The heat map illustrates diferent levels of gene expression, and the KEGG data show upregulation of the Hippo signaling pathway in FGF23-treated cells (significant enrichment at *P* < 0.05, Fisher's exact test). **(C)** Cells were treated with recombinant FGF23 for different time. Representative immunoblots and densitometric data show that FGF23 induces YAP phosphorylation. **(D)** AVICs were treated with PRN1371 prior to FGF23 stimulation for 2 hours. Representative immunoblots and densitometric data show that global FGFR inhibitor suppresses FGF23-induced YAP phosphorylation. **(F)** Cells were treated with verteporfin (200 nM) for 2 hours prior to FGF23 stimulation. Representative immunoblots and densitometric data show that inhibition of YAP reduces inflammatory, fibrogenic, and osteogenic responses following FGF23 stimulation. Quantitative data are expressed as mean ± SEM. n = 3 or 4 cell isolates from distinct donor valves in each group. *P < 0.05 versus control and ^#^*P* < 0.05 versus FGF23 alone.

We examined the expression of FGFR1 and FGFR4 in human AVICs and confirmed their presence. Interestingly, an exposure to FGF23 for varied time (24, 48 or 72 hours) elevated the levels of both of the two FGFR isoforms (**Supplementary Figure 3A** and** B**). To determine whether FGF23 induces the pathobiological changes in AVICs through FGFR, we treated human AVICs with global FGFR inhibitor (PRN1371). As shown in **Figure 4A and Supplementary Figure 4**, global inhibition of FGFR attenuated FGF23-induced inflammatory, fibrogenic and osteogenic responses in AVICs. Subsequently, we treated human AVICs with ponatinib (strong inhibition of FGFR1 at a low concentration) and BLU-554 (FGFR4 inhibitor) to determine the relative roles of FGFR1 and FGFR4 in mediating the FGF23 effects. The data presented in **Supplementary Figure 4** indicate that FGF23 induces the inflammatory and fibrogenic responses in AVICs mainly through FGFR1 and provokes the osteogenic response via FGFR4.

We performed a systematic analysis using total RNA-sequencing technology to elucidate the mechanism by which FGF23 induces the pathobiological changes in AVICs. Heatmap data displayed distinct gene expression profiles in FGF23-treated and untreated cells. Venn diagrams identified 1,182 genes shared between control AVICs and those exposed to FGF23, with 507 genes unique to control AVICs and 681 genes specific to FGF23-treated AVICs (**Supplementary Figure 5**). Moreover, KEGG enrichment analysis revealed that the Hippo signaling pathway is markedly upregulated in AVICs exposed to FGF23 (**Figure 4B** and **Supplementary Figure 5**).

YAP serves as a key downstream mediator of the Hippo signaling pathway 25. We assessed effect of FGF23 on YAP phosphorylation in human AVICs. As shown in **Figure 4C**, YAP phosphorylation was increased, peaking at 2 hours following FGF23 stimulation. To examine whether FGF23 activates YAP through FGFR, we treated human AVICs with an FGFR inhibitor for 2 hours prior to FGF23 stimulation for 2 hours. Inhibition of FGFR suppressed FGF23-induced YAP phosphorylation (**Figure 4D**). To evaluate the role of YAP in mediating the effects of FGF23 on AVICs, we applied a YAP inhibitor (verteporfin) to AVICs prior to the exposure to FGF23. Verteporfin dose-dependently suppressed YAP phosphorylation in AVICs (**Supplementary Figure 6**), and inhibition of YAP with verteporfin (200 nM) attenuated the inflammatory, fibrogenic and osteogenic responses in AVICs exposed to FGF23 (**Figure 4E**).

### Klotho interacts with FGF23 to mitigate AVIC fibrosis and calcification

**Figure 5:**
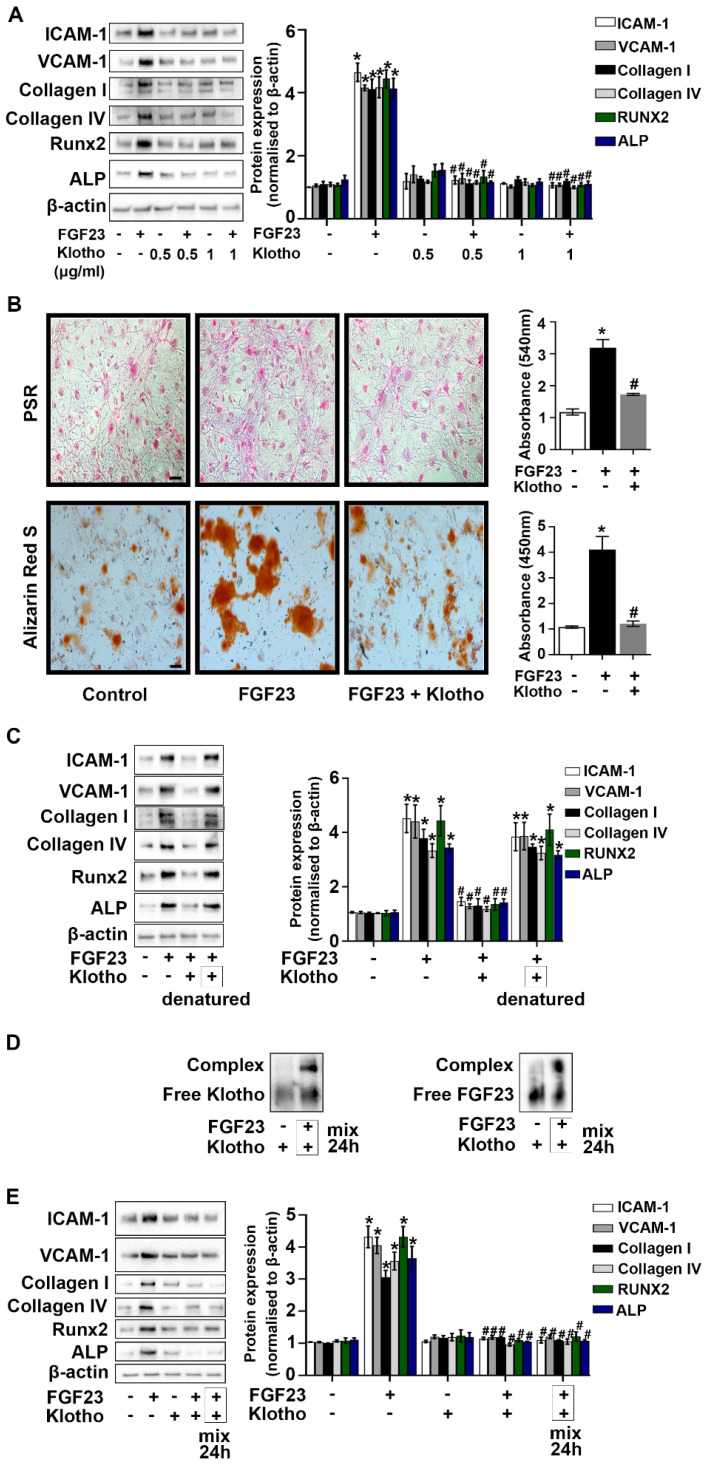
**Klotho interacts with FGF23 to suppress its effects in AVICs. (A)** Human AVICs from normal valves were treated with varied concentrations of recombinant Klotho prior to the exposure to recombinant FGF23 for 72 hours. Representative immunoblots and densitometric data show that Klotho downregulates the effect of FGF23 in inducing inflammatory, fibrogenic and osteogenic mediators. **(B)** Representative images of Picrosirius Red (PSR) staining, Alizarin Red S staining and corresponding spectrophotometric data normalized by cell density show that recombinant Klotho suppresses collagen and calcium deposition in cells following prolonged exposure to FGF23. Original magnification 10x. Scale bar = 100 µm. **(C)** Representative immunoblots and densitometric data show that heat-denatured Klotho has no effect on FGF23-induced expression of inflammatory, fibrogenic and osteogenic mediators. **(D)** Recombinant FGF23 was incubated with recombinant Klotho for 24 hours. Representative immunoblot probed with anti-Klotho or anti-FGF23 identified corresponding free protein and the formation of complexes between these two proteins. **(E)** Pre-mixed Klotho-FGF23 has minimal effects on the expression of inflammatory, fibrogenic and osteogenic mediators in AVICs. Quantitative data are expressed as mean ± SEM. n = 3 or 4 cell isolates from distinct donor valves in each group. *P < 0.05 versus control and ^#^*P* < 0.05 versus FGF23 alone.

We knocked-down Klotho protein in AVICs to evaluate the relationship between Klotho and FGF23. As shown in **Supplementary Figure 7**, FGF23 protein levels were increased in the cells with Klotho knockdown. To determine the impact of Klotho on FGF23's effect in AVICs, we treated cells with recombinant Klotho prior to FGF23 exposure for 72 hours. Recombinant Klotho (0.5 and 1.0 μg/mL) suppressed AVIC inflammatory, fibrogenic, and osteogenic responses (**Figure 5A**). In addition, recombinant Klotho reduced collagen and calcium deposition in AVICs exposed to FGF23 (**Figure 5B**). We then assessed the impact of denatured recombinant Klotho (boiling in water for 30 minutes) on FGF23 effects in AVICs. Denaturation of Klotho protein resulted in the loss of its ability to suppress FGF23 effects in AVICs (**Figure 5C**). Further, incubation of FGF23 with recombinant Klotho resulted in the formation of complexes of these two proteins (**Figure 5D**) and markedly decreased the effects of FGF23 in AVICs (**Figure 5E**).

### Treatment of CKD mice with recombinant Klotho attenuates aortic valve lesions

**Figure 6:**
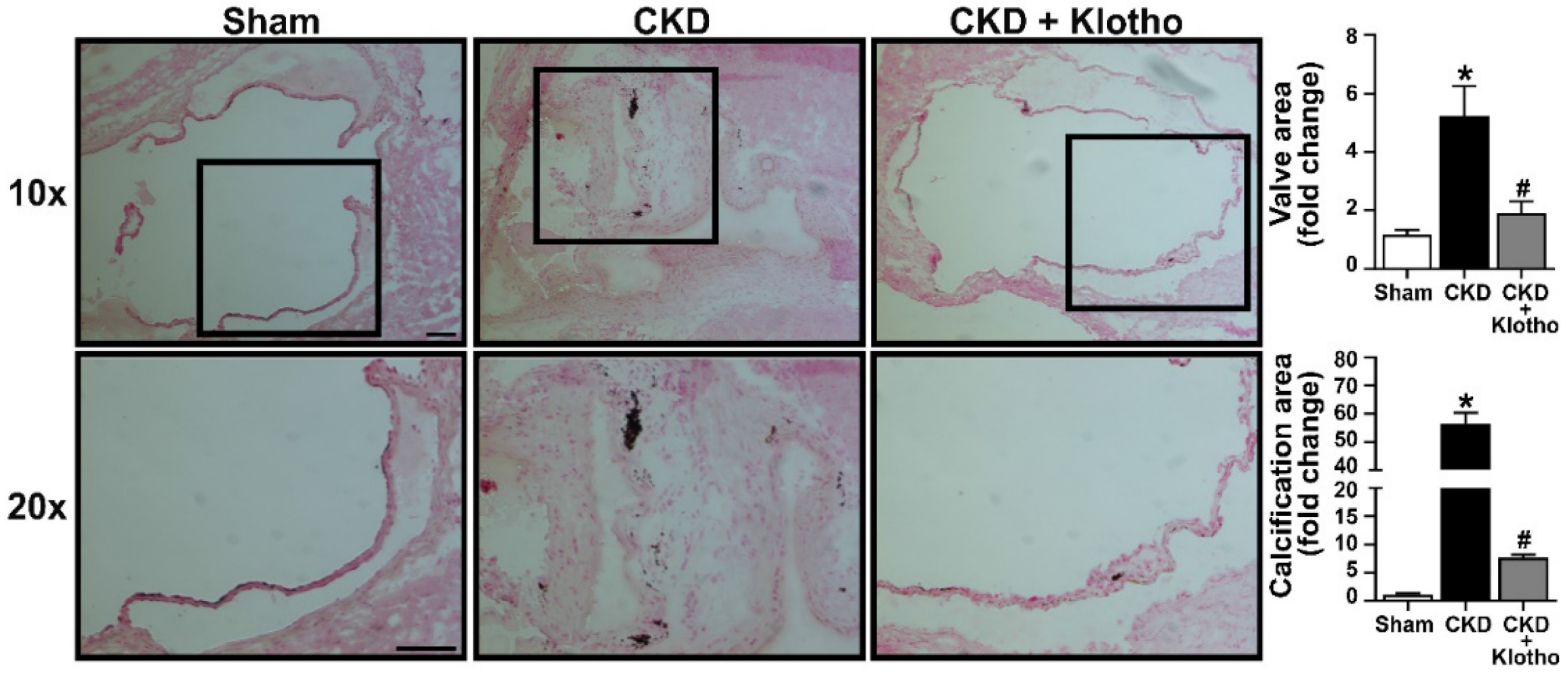
** Recombinant Klotho attenuates aortic valve thickening and calcification in mice subjected to CKD protocol.** Old mice subjected to CKD protocol were continuously treated with recombinant Klotho (20 µg/kg/day) in the period of week 3 to 6 using a subcutaneous osmotic pump. Representative images show that treatment with recombinant Klotho reduced aortic valve thickening and calcification. Scale bar = 100 μm. Values are mean ± SEM. n = 4 mice per group. *P < 0.05 versus sham and ^#^*P* < 0.05 versus CKD.

To evaluate the potential of recombinant Klotho in attenuating aortic valve fibrosis and calcification in CKD mice, we administered recombinant Klotho to CKD-developing mice at 20 μg/kg/day using osmotic pumps. Aortic valves were collected at the end of the treatment and examined with von Kossa staining. As shown in **Figure 6**, treatment with recombinant Klotho reduced aortic valve thickening and calcification in mice subjected to CKD protocol. This finding suggest that recombinant Klotho has the potential for alleviating aortic valve lesions induced by CKD.

## Discussion

CAVD, characterized by fibrosis and calcification in valvular leaflets, is prevalent in the elderly (65 years and older), particularly those with CKD. Progressive fibrosis and calcification cause valvular thickening and dysfunction, resulting in aortic stenosis and heart failure. Currently, surgical or interventional valve replacement is the sole treatment for aortic stenosis, with a large number of cases posing complications. Thus, it is important to develop pharmacological treatments for prevention of CAVD progression to aortic stenosis. However, the mechanism underlying CAVD pathogenesis remains incompletely understood. In this study, we demonstrate that: (1) CKD upregulates FGF23 level and exacerbates Klotho insufficiency in aortic valves of old mice, leading to aortic valve lesions; (2) FGF23 elevates the pathobiological activities, including inflammatory, fibrogenic and osteogenic activities, in human AVICs through the FGFR-YAP signaling pathway; (3) soluble Klotho suppresses the effect of FGF23 on AVIC pathobiological activities through molecular interaction with FGF23; and (4) recombinant Klotho is capable of reducing aortic valve lesions. These findings underscore the critical role of FGF23 overproduction and Klotho insufficiency in promoting aortic valve fibrosis and calcification in CKD mice. Furthermore, these findings highlight the therapeutic potential of Klotho for prevention of aortic valve lesion progression in old subjects with CKD.

Clinical studies have reported greater CAVD prevalence and its exaggerated progression in old adults with CKD 8,26. We developed an old mouse model of CKD in order to better understand how CKD induces CAVD and promotes its progression. Using this CKD model, we observed that aortic valve thickening and calcification nodule formation are associated with elevated FGF23 levels in both plasma and aortic valve tissue of CKD mice. While FGF23 regulates phosphate and vitamin D metabolism, elevated levels of FGF23 are associated with cardiovascular events and adverse outcomes in patients with CKD 17. It is likely that FGF23 plays a role in the pathogenesis of CAVD in subjects with CKD.

In multiple experiments using human AVICs, we found that FGF23 induces the upregulation of two essential adhesion molecules, ICAM-1 and VCAM-1 that can initiate the recruitment of immune cells and trigger an inflammatory cascade in the aortic valve. FGF23 also enhances the expression of collagen I and collagen IV and induces collagen deposition, an *in vitro* indicator of fibrogenesis. Apparently, FGF23 is pro-fibrogenic in aortic valve cells. Furthermore, FGF23 induces the expression of osteoblastic biomarkers and promotes calcium deposition in AVICs, contributing to the mechanism of aortic valve calcification. The suppressive effects of FGF23 knockdown and neutralization on the spontaneous inflammatory, fibrogenic and osteogenic activities in AVICs of diseased valves support the notion that FGF23 is a critical mediator of aortic valve inflammation, fibrosis and calcification in CAVD associated with CKD.

FGF23 interactions with FGFR to modulate tissue pathobiology 27. FGFR isoforms have been reported to play important roles in mediating inflammation and fibrosis in several diseases 28-32. In the current study, we observed that human AVICs express FGFR1 and FGFR4. It is noteworthy that FGF23 appears to utilize different FGFR isoforms to induce specific cellular responses in AVICs. In this regard, our observations indicate that FGFR1 plays an important role in mediating the inflammatory and fibrogenic responses to FGF23 in human AVICs while FGF23 utilizes FGFR4 to provoke the osteogenic response. However, other FGFR isoform(s) may be also involved in mediating the effect of FGF23 on the inflammatory and fibrogenic activities in human AVICs since ponatinib is not a highly selective FGFR1 inhibitor. Nevertheless, selective blockade of different FGFR isoform may suppress specific pathobiological activity in the aortic valve in the setting of CKD-induced CAVD.

FGF23 can activate multiple signaling pathways via FGFR 33. Using a systemic approach, we uncovered that FGF23 activates the Hippo-YAP signaling pathways in human AVICs. This signaling pathway mediates cell proliferation and differentiation and has been reported to be involved in the mechanism of tissue fibrosis associated with pathological conditions 34,35. In the current study, we found that FGF23 induces YAP phosphorylation, an indicator of YAP activation, via FGFR as global FGFR inhibitor abolished FGF23-induced YAP phosphorylation. More importantly, inhibition of YAP with a specific inhibitor markedly attenuated the potency of FGF23 in the upregulation of pathobiological activities in human AVICs. Thus, the YAP signaling pathway plays a major role in mediating the effects of FGF23 on AVIC pathobiological activities involved in CAVD pathogenesis. It should be noted that we observed that the TGF-β pathway is also upregulated in AVICs exposed to FGF23. Related to this observation, a previous study reported that FGF23 induces the phosphorylation of SMAD2/3, a critical mediator of TGF-β signaling 36. As the SMAD signaling pathway contributes to the development of CAVD 37,38. Studies are needed to evaluate the relative role of the TGF-β/SMAD signaling pathway in the mechanism underlying FGF23-induced valvular cell fibrogenesis and calcification.

Aortic valve tissue and cells of patients with aortic stenosis have reduced levels of anti-aging protein Klotho 19. Several studies reported that reduced serum Klotho levels might be a risk factor for coronary artery calcification in CKD patients 40,41. In the current study, a significant decrease in Klotho levels was observed in the plasma and aortic valves of CKD mice. Our *in vivo* experiments reveal that administration of recombinant Klotho attenuates CKD-induced aortic valve lesions, resulting in reduced aortic valve thickening and calcification in old mice subjected to CKD protocol. The effect of Klotho on aortic valve thickening and calcification is substantiated by its suppression of collagen and calcium deposition in human AVICs subjected to prolonged exposure to FGF23. Further, recombinant Klotho attenuates the potency of FGF23 in upregulation of the inflammatory, fibrogenic and osteogenic activities in human AVICs. These findings align with our prior findings that recombinant Klotho suppresses inflammatory and osteogenic activities induced by pro-inflammatory mediators in aortic valve cells 10,19, and are in agreement with previous studies showing that Klotho can mitigate vascular smooth muscle cell calcification 39,40. Together, the findings of the current study suggest that Klotho inhibits the effects of FGF23 to mitigate CKD-induced aortic valve lesions.

The results of further experiments indicate that Klotho interacts with FGF23 to reduce its potency in upregulating AVIC inflammatory, fibrogenic and osteogenic activities. This notion is supported by three observations. First, heat-denatured Klotho has no effect on FGF23 potency on AVIC inflammatory, fibrogenic and osteogenic activities. Second, incubation of a mix of recombinant Klotho and recombinant FGF23 results in the formation of protein-protein complexes. Lastly, recombinant FGF23 pre-mixed with recombinant Klotho essentially losses its effect on AVIC inflammatory, fibrogenic and osteogenic activities. It appears that Klotho is capable of binding to FGF23 to form molecular complexes, and the capture of FGF23 by Klotho prevents FGF23 action on AVICs. It appears that recombinant Klotho is druggable for mitigation of FGF23-mediated pathobiology activity in subjects with CKD.

Studies have reported that the administration of recombinant Klotho to mice with CKD resulted in decreased FGF23 plasma levels, while Klotho-deficient mice have higher levels of FGF23 in the circulation 41. We observed that Klotho knockdown in human AVICs increases cellular production of FGF23. Thus, Klotho insufficiency in CKD mice appears to be a mechanism of elevating FGF23 levels in the circulation and aortic valve tissue. Conversely, elevation of Klotho levels by administration of recombinant Klotho would suppress FGF23 production. As Klotho has the potential to modulate FGF23 levels, a reduction in FGF23 could be an additional mechanism contributing the beneficial effects of recombinant Klotho in reducing CKD-induced aortic valve lesions.

Aging may induce CKD-associated CAVD through mechanisms independent of Klotho insufficiency, including the induction of cellular damage, modulation of gene expression and attenuation of tissue regenerative/repairment capacities 42. Further studies are needed to investigate how aging promotes CAVD development through Klotho-independent mechanisms. Nevertheless, the reduced levels of Klotho observed in old CKD mice contribute to the mechanism underlying CKD-induced aortic valve lesions due to upregulation of FGF23 levels and causing inadequate capacity of antagonizing FGF23 action.

It should be noted that AVICs from diseased valves and normal AVICs exposed to FGF23 have greater inflammatory activities. A number of studies demonstrate the inflammatory mechanisms in CAVD pathobiology 43. In this regard, inflammatory infiltrates are present in calcified areas diseased aortic valves 44, and pro-inflammatory cytokines, such as TNF-α and IL-1β, have been found to upregulate the expression of osteogenic mediators in AVICs 45,46. Our studies revealed that human AVICs express functional Toll-like receptors 47. Activation of Toll-like receptors 2, 3 or 4 induces the production of pro-inflammatory mediators that activate AVICs to differentiate into myofibroblastic or osteoblast-like cells, resulting in valvular fibrosis and calcification 47-50. Thus, dysregulated inflammatory activity in aortic valve tissue plays a mechanistic role in CAVD development and progression. It is likely that upregulation of inflammatory activity in AVICs is an independent mechanism by which FGF23 promotes aortic valve thickening and calcification.

One of the limitations of this study is its relatively small sample size. To enhance the robustness of the findings, all *in vitro* experiments were conducted using AVIC isolates from different donors having diseased valves or normal valves. Further study using larger sample size could provide more convincing results. Additionally, the cultured human AVICs utilized in this study may exhibit unique characteristics compared to cells in their natural *in vivo* environment. This may limit the implication of all of the *in vitro* findings to the *in vivo* setting.

## Conclusion

Overall, the current study demonstrates that CKD induces Klotho insufficiency and FGF23 accumulation, and both of these two changes contribute to the mechanism underlying CAVD development and progression. FGF23 induces fibrosis and calcification in human AVICs through FGFR/YAP signaling. Klotho interacts with FGF23 to suppress its pro-pathobiological effects in human AVICs and is capable of reducing aortic valve lesions in old CKD mice. These novel findings suggest a novel role of FGF23 in the pathogenesis of CAVD associated with CKD and point to the therapeutic potential of Klotho for prevention of CAVD development and progression in old CKD subjects with high risks for CAVD and aortic stenosis.

## Supplementary Material

Supplementary figures.

## Abbreviations

ALP: alkaline phosphatase
AVICs: aortic valve interstitial cells
CAVD: calcific aortic valve disease
CKD: chronic kidney disease
DAMPs: damage-associated molecular patterns
DAPI: diamidino-2-phenylindole
FBS: fetal bovine serum
FGF23: fibroblast growth factor 23
FGFR: fibroblast growth factor receptor
H&E: hematoxylin and eosin
ICAM-1: intercellular adhesion molecule 1
PBS: phosphate-buffered saline
PSR: Picrosirius Red
RUNX2: Runt-related transcription factor 2
shRNA: small hairpin RNA
VCAM-1: vascular cell adhesion molecule 1
WGA: wheat germ agglutinin
YAP: Yes-associated protein
