# Supplementary material for: Molecular Interaction of Soluble Klotho with FGF23 in the Pathobiology of Aortic Valve Lesions Induced by Chronic Kidney Disease
Source: Int J Biol Sci. 2024 Jun 17;20(9):3412–25. doi: 10.7150/ijbs.92447 (PMC11234222; doi:10.7150/ijbs.92447)
Supplement: Supplementary file 1 — Supplementary figures. [file ijbsv20p3412s1.pdf]

Supplementary Figures

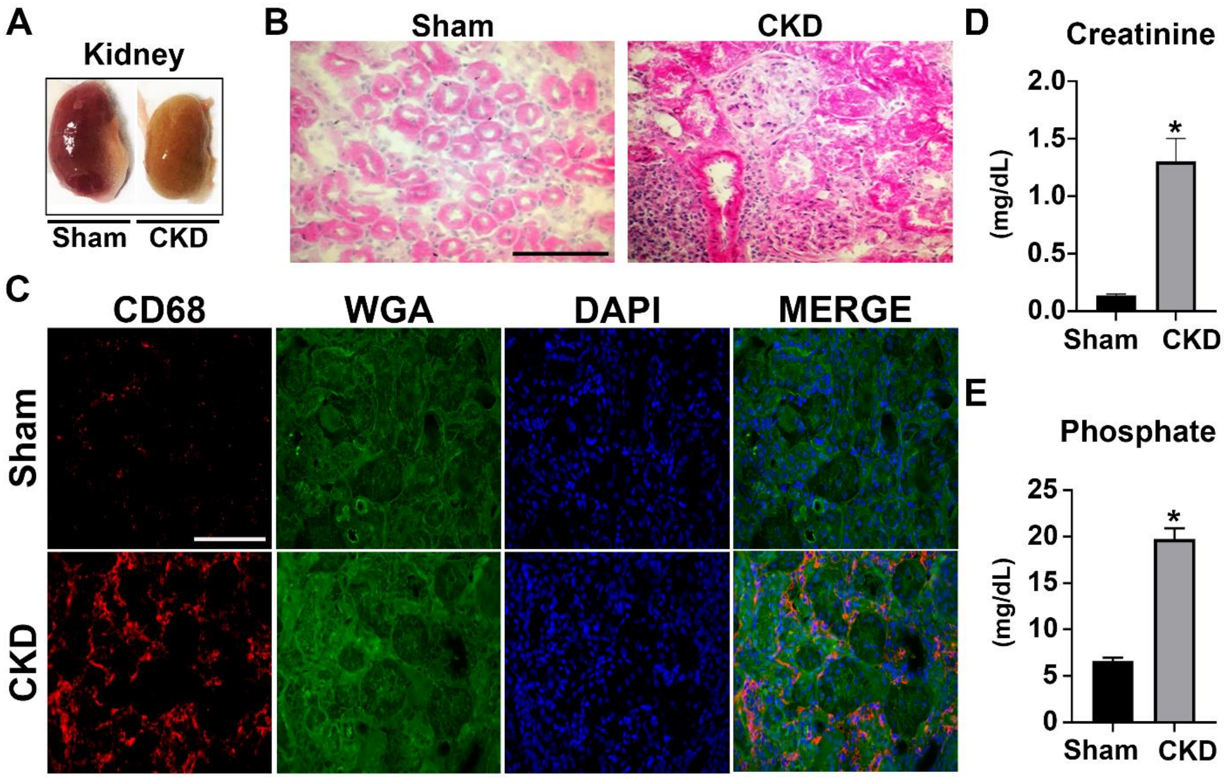

Supplementary Figure 1. Old mouse model of chronic kidney disease. (A) Old C57 BL/6 mice (18-20 months old) were fed with an AIN-76A diet (high in calcium and phosphate) and given adenine (0.8 mg/day) and calcitriol (0.25 µg/kg/day). Representative images of the kidney in sham and chronic kidney disease (CKD) mice are shown. **(B** and **C)** Representative images of HE and CD68 (monocytes/macrophages biomarker, red) staining of kidney section of sham and CKD mice showing tubular damages and monocytes/macrophages accumulation. Original magnification 40x. Scale bar: 100 µm. **(D** and **E)** Plasma creatinine and phosphate levels in sham and CKD mice, indicating renal dysfunction. n = 3 mice per group. Values are mean ± SEM. *$P < 0.05$ vs sham.

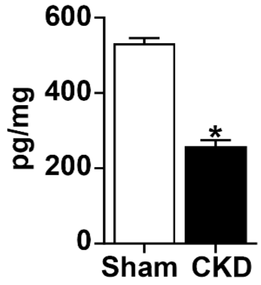

Supplementary Figure 2. Kidney Klotho levels are lower in CKD mice. Klotho protein levels were assessed using an ELISA kit. The data show a significant reduction in Klotho levels in the kidney tissue of CKD mice. n = 4 mice per group. Values are mean ± SEM. *$P < 0.05$ vs sham.

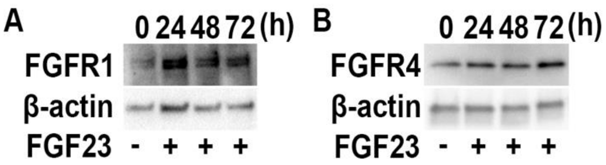

Supplementary Figure 3. FGFR1 and FGFR4 are present in human AVICs. Human AVICs were treated with recombinant FGF23 for 24 to 72 hours. Representative immunoblots show that FGFR isoforms 1 and 4 are present in human AVICs with or without FGF23 treatment, and their levels are increased in cells exposed to FGF23.

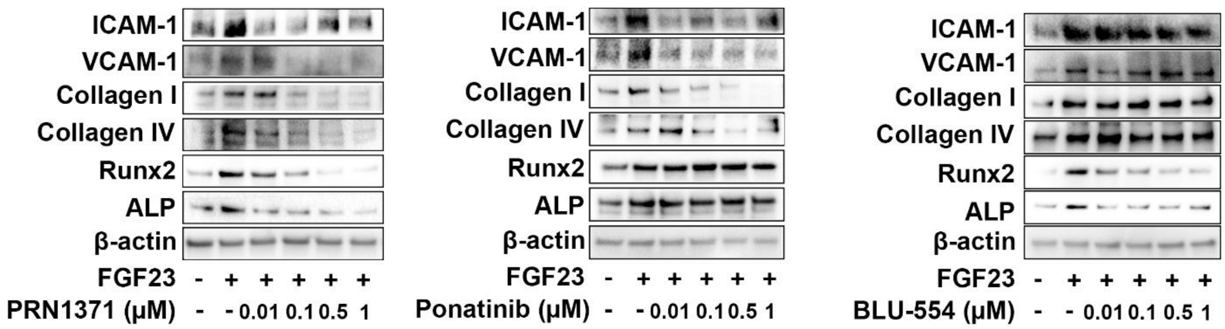

Supplementary Figure 4. FGFR1 and FGFR4 appear to have differential roles in elevating AVIC inflammatory, fibrogenic and osteogenic activities. Human AVICs were pre-treated with different concentrations of PRN1371, ponatinib or BLU-554 for 2 hours followed by FGF23 (40 ng/mL) stimulation for 72 hours. Representative immunoblots show that inhibition of FGFR1 using ponatinib attenuates the inflammatory and fibrogenic responses to FGF23, and inhibition of FGFR4 with BLU-554 suppresses the osteogenic response to FGF23.

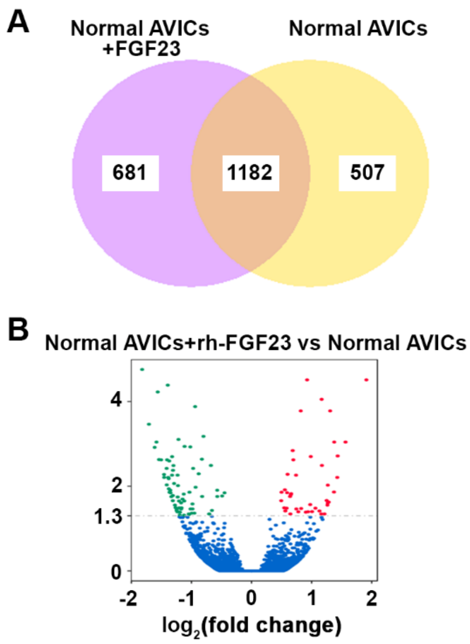

Supplementary Figure 5. RNA sequencing reveals distinct gene clusters and expression profiles in human AVICs incubated in the presence and absence of recombinant FGF23. (**A**) Venn diagrams reveal shared gene expression between normal AVICs and FGF23-treated normal AVICs, encompassing 1,182 genes. Additionally, untreated normal AVICs displayed 507 unique genes, while FGF23-treated AVICs exhibited 681 specific genes. (**B**) Volcano plot illustrates FGF23-induced gene expression alteration in AVICs, with 43 upregulated genes (in red) and 77 downregulated genes (in green). n = 4 distinct donors per group.

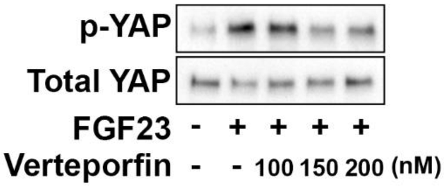

Supplementary Figure 6. Verteporfin suppresses YAP phosphorylation induced by FGF23. AVICs were subjected to various concentrations of verteporfin for 2 hours followed by FGF23 stimulation for 2 hours. Verteporfin dose-dependently suppresses FGF23-induced YAP phosphorylation.

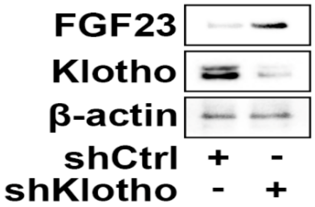

Supplementary Figure 7. Klotho knockdown enhances FGF23 expression. Human AVICs were treated with lentivirus expressing Klotho shRNA (100 nmol/L) for 72 hours. Representative immunoblots show that knockdown of Klotho increases FGF23 protein level. Ctrl = control.
